# An improved assembly of the pearl millet reference genome using Oxford Nanopore long reads and optical mapping

**DOI:** 10.1093/g3journal/jkad051

**Published:** 2023-03-09

**Authors:** Marine Salson, Julie Orjuela, Cédric Mariac, Leïla Zekraouï, Marie Couderc, Sandrine Arribat, Nathalie Rodde, Adama Faye, Ndjido A Kane, Christine Tranchant-Dubreuil, Yves Vigouroux, Cécile Berthouly-Salazar

**Affiliations:** DIADE, Université de Montpellier, Institut de Recherche pour le Développement (IRD), Centre de coopération internationale en recherche agronomique pour le développement (CIRAD), 34394 Montpellier, France; DIADE, Université de Montpellier, Institut de Recherche pour le Développement (IRD), Centre de coopération internationale en recherche agronomique pour le développement (CIRAD), 34394 Montpellier, France; DIADE, Université de Montpellier, Institut de Recherche pour le Développement (IRD), Centre de coopération internationale en recherche agronomique pour le développement (CIRAD), 34394 Montpellier, France; DIADE, Université de Montpellier, Institut de Recherche pour le Développement (IRD), Centre de coopération internationale en recherche agronomique pour le développement (CIRAD), 34394 Montpellier, France; DIADE, Université de Montpellier, Institut de Recherche pour le Développement (IRD), Centre de coopération internationale en recherche agronomique pour le développement (CIRAD), 34394 Montpellier, France; Centre National de Ressources Génomiques Végétales (CNRGV), Institut national de recherche pour l’agriculture, l’alimentation et l’environnement (INRAE), 31320 Castanet-Tolosan, France; Centre National de Ressources Génomiques Végétales (CNRGV), Institut national de recherche pour l’agriculture, l’alimentation et l’environnement (INRAE), 31320 Castanet-Tolosan, France; Centre d’Etude Régional pour l’Amélioration de l’Adaptation à la sécheresse (CERAAS), Institut Sénégalais de Recherches Agricoles (ISRA), BP 3320 Thiès, Sénégal; Laboratoire Mixte International Adaptation des Plantes et microorganismes associés aux Stress Environnementaux (LMI LAPSE), IRD/ISRA/UCAD, BP 1386, CP 18524 Dakar, Sénégal; Centre d’Etude Régional pour l’Amélioration de l’Adaptation à la sécheresse (CERAAS), Institut Sénégalais de Recherches Agricoles (ISRA), BP 3320 Thiès, Sénégal; Laboratoire Mixte International Adaptation des Plantes et microorganismes associés aux Stress Environnementaux (LMI LAPSE), IRD/ISRA/UCAD, BP 1386, CP 18524 Dakar, Sénégal; DIADE, Université de Montpellier, Institut de Recherche pour le Développement (IRD), Centre de coopération internationale en recherche agronomique pour le développement (CIRAD), 34394 Montpellier, France; DIADE, Université de Montpellier, Institut de Recherche pour le Développement (IRD), Centre de coopération internationale en recherche agronomique pour le développement (CIRAD), 34394 Montpellier, France; DIADE, Université de Montpellier, Institut de Recherche pour le Développement (IRD), Centre de coopération internationale en recherche agronomique pour le développement (CIRAD), 34394 Montpellier, France

**Keywords:** pearl millet, assembly, Oxford Nanopore long reads, optical mapping

## Abstract

Pearl millet (*Pennisetum glaucum* (L.)) R. Br. syn. *Cenchrus americanus* (L.) Morrone) is an important crop in South Asia and sub-Saharan Africa which contributes to ensuring food security. Its genome has an estimated size of 1.76 Gb and displays a high level of repetitiveness above 80%. A first assembly was previously obtained for the Tift 23D2B1-P1-P5 cultivar genotype using short-read sequencing technologies. This assembly is, however, incomplete and fragmented with around 200 Mb unplaced on chromosomes. We report here an improved quality assembly of the pearl millet Tift 23D2B1-P1-P5 cultivar genotype obtained with an approach combining Oxford Nanopore long reads and Bionano Genomics optical maps. This strategy allowed us to add around 200 Mb at the chromosome-level assembly. Moreover, we strongly improved continuity in the order of the contigs and scaffolds within the chromosomes, particularly in the centromeric regions. Notably, we added more than 100 Mb around the centromeric region on chromosome 7. This new assembly also displayed a higher gene completeness with a complete BUSCO score of 98.4% using the Poales database. This more complete and higher quality assembly of the Tift 23D2B1-P1-P5 genotype now available to the community will help in the development of research on the role of structural variants and more broadly in genomics studies and the breeding of pearl millet.

## Introduction

Pearl millet (*Pennisetum glaucum* (L.)) R. Br. syn. *Cenchrus americanus* (L.) Morrone) is a cereal adapted to high temperature and is mainly cultivated in sub-Saharan Africa and South Asia. It is the staple food for more than 90 million farmers, and research projects aiming to improve this crop's productivity and resilience may thus contribute to greater food security. Obtaining a more complete pearl millet reference genome assembly and improving its quality will help us to better carry out genetic and genomic studies of this important crop.

Next-generation sequencing technologies such as Illumina technology enabled the acquisition of a large number of genomes in the 2010s, including nonmodel species both in the animal and plant kingdoms. A genome for pearl millet was assembled and published in 2017 (Varshney *et al*. 2017) using the inbred Tift 23D2B1-P1-P5 cultivar genotype as the reference (BioSample identifier: SAMN04124419). Pearl millet is a cross-pollinated diploid with seven chromosomes (2*n* = 14). Its genome size was estimated at 1.76 Gb with more than 80% repetitive sequences (Varshney *et al*. 2017). However, around 200 Mb remained unplaced in the pearl millet reference genome (Varshney *et al*. 2017) and the chromosomes were fragmented and displayed a high Ns content above 13% (GCA_002174835, European Nucleotide Archive).

Assembly of large and complex genomes obtained with short-read sequencing technologies is often incomplete and fragmented (Belser *et al*. 2018). Combining long-read sequencing and optical mapping has proven to be an effective approach to improve the quality of assemblies of complex plant genomes over the last few years (Belser *et al*. 2018, Istace *et al*. 2021, Belser *et al*. 2021, Aury *et al*. 2022). Recent studies performed with genomes of higher quality have highlighted the importance of structural variations such as inversions in the evolution and adaptation of species (Wellenreuther and Bernatchez 2018, Huang and Rieserberg 2020). A high-quality reference genome is, however, required to detect and study such variants. To improve the quality of the Tift 23D2B1-P1-P5 genome, we therefore generated Bionano Genomics optical maps and long reads obtained by Oxford Nanopore Technologies (ONT) sequencing. The combined use of these two types of data allowed us to improve the N50 of scaffolds by two orders of magnitude with a N50 of 86 Mb, and we added around 200 Mb at the chromosome-level assembly. The improvement of the quality of the assembly was also verified by comparing the chromosomes of both the new and the previous genomes with the optical maps obtained for a control line PMiGAP257/IP-4927. The comparison highlighted the better continuity in the order of the contigs and the scaffolds of the new assembly, notably in the centromeric regions. This assembly will thus allow more efficient identification of structural variants in pearl millet populations and a better understanding of the genomics of this important crop.

## Materials and methods

### Plant materials and sequencing

Biological material for both accessions was obtained from ICRISAT in Niamey. For Tift 23D2B1-P1-P5, genotyping of 14 SSRs was used to ensure the homozygosity of the individual extracted. PMiGAP257/IP-4927 is an inbred line from a Senegalese sauna pearl millet. High-molecular weight DNA extraction was performed using a previously published protocol (Mariac *et al*. 2019, https://dx.doi.org/10.17504/protocols.io.83shyne). Briefly, the isolation of plant nuclei is performed from 1 gram of fresh young leaves previously ground in liquid nitrogen. The isolated nuclei are then lysed (MATAB) and the DNA is purified with chloroform/isoamyl alcohol (24 : 1) and then precipitated with isopropanol. All transfer steps were performed with a pipette tip cut at the extremity and homogenization steps were performed by slow inversion to limit mechanical shearing of the DNA molecules. DNAs were quantified by fluorometry (Qubit) and qualitatively assessed using pulsed field electrophoresis to ensure that fragment sizes ranged from 40 to 150 kb. Oxford Nanopore DNA library preparation (SQKLSK109-PromethION, Genomic DNA ligation protocol) and sequencing were performed by Novogen Co. LTD.

### Long-read ONT assembly and polishing

The different steps of the assembly are summarized in Supplementary Fig. 1. Base calling on Oxford Nanopore Technologies (ONT) reads was performed with guppy (v. 6.0.6 and the dna_r9.4.1_450bps_hac_prom.cfg model). Reads shorter than 5 kb and with a quality score below 10 were excluded with NanoFilt (v. 1.0, De Coster *et al*. 2018). The ONT assembly was performed with filtered reads using the CulebrONT pipeline (Orjuela *et al*. 2022, v 2.1.0) and Flye assembler (v. 2.9, Kolmogorov *et al*. 2019). Two rounds of Racon (v. 1.5.0, Vaser *et al*. 2017) and Medaka (v. 1.6.1, https://github.com/nanoporetech/medaka) were also used to polish and correct the contigs using the ONT reads. The ONT contigs were finally polished with high-quality Illumina short reads using Hapo-G (v 1.3, Aury and Istace 2021). The short reads from the same Tift 23D2B1-P1-P5 genotype (175 Gb of raw data corresponding to 97X coverage, NCBI SRA accession SRP063925, and the list of SRR identifiers used: SRR2489264-SRR2489273, Varshney *et al*. 2017) were trimmed with cutadapt (v3.1, -m 35, -q 30,30 parameters, Martin 2011) and aligned to the ONT contigs using bwa-mem2 (v 2.2.1, Li and Durbin 2010, Vasimuddin *et al*. 2019) with -I 210,100,500,100 parameters to handle two different insert sizes in the short reads paired-end libraries of 170 and 250 bases. Only properly paired reads were kept using the software samtools (v. 1.9, -f 0 × 02 parameter, Danecek *et al*. 2021) and two rounds of short reads correction with Hapo-G (v 1.3, Aury and Istace 2021) were performed with default parameters (Aury and Istace 2021).

Purge Haplotigs (Roach *et al*. 2018) was used in order to identify potential false duplications in the assembly. The long reads were aligned to the ONT contigs with minimap2 (v. 2.24, Li H. 2018) and the hist command of Purge Haplotigs (v. 1.1.1, Roach *et al*. 2018) was launched to obtain an assembly-wide read depth histogram.

### Optical mapping data generation and comparison with the old pearl millet reference genome

Ultra-HMV DNA extraction and optical map generation were carried out using the Bionano Prep Plant tissue DNA Isolation and Bionano Prep Direct Label and Stain Label (DLS) protocols and were performed by the French Plant Genomic Resources Centre (CNRGV) of the French National Research Institute for Agriculture, Food and Environment (INRAE). Optical mapping data were generated for the Tift 23D2B1-P1-P5 and the PMiGAP257/IP-4927 genotypes with the Bionano Genomics Saphyr system. The DLE-1 enzyme and the Direct Label and Stain technology were used. Molecules smaller than 150 kb and with fewer than nine labels were excluded. De novo assembly was performed with the filtered molecules using Bionano Solve pipeline (v. 3.5.1, Shelton *et al*. 2015).

The seven chromosomes of the old pearl millet reference genome (Varshney *et al*. 2017, GCA_002174835.1) were converted into optical maps using the *fa2cmap_multi_color.pl* script of Bionano Solve (v3.3, Shelton *et al*. 2015) and were aligned with the Tift 23D2B1-P1-P5 assembled optical maps using the *runCharacterize.py* script of Bionano Solve with RefAligner and the default parameters (v3.3, Shelton *et al*. 2015, Yuan *et al*. 2020). Alignments were visualized with Bionano Access (v 3.7, Yuan *et al*. 2020) and the cumulative size of the optical maps assigned to each chromosome was calculated. The optical maps were assigned to the chromosome with which they shared the longest aligned region. We calculated the Pearson correlation coefficient between the old reference chromosome lengths and the cumulative sizes of the optical maps aligned to each chromosome with the R function cor.test() (R version 4.2.1) and visually inspected the correlation using the function geom_smooth() of the R package ggplot2 (v. 3.3.6, Wickham H 2016).

### Hybrid scaffolding with optical maps

Hybrid scaffolding was performed with both the ONT contigs and the assembled optical maps of the Tift 23D2B1-P1-P5 genotype using Bionano Solve (v. 3.3, *hybridScaffold.pl* script with -B 2 -N 2 parameters, Shelton *et al*. 2015). We then used the Bionano Scaffolding Correction Tool (BiSCoT v. 2.3.3, Istace *et al*. 2020) with the default parameters in order to remove artefactual duplications from the hybrid scaffolds. TGS Gap-Closer (v. 1.2.0, Mengyang Xu *et al*. 2020) was used to perform gap filling and reduce the total number of Ns in the hybrid scaffolds. This step may also correct the lack of Bionano precision in predicting the size of gaps below 10 kb (Mengyang Xu *et al*. 2020). We only used ONT reads with a quality score *Q* > 12 and length greater than 10 kb. Additionally, we corrected these ONT long reads using Illumina high-quality short reads with Hapo-G (v 1.3, Aury and Istace 2021). TGS Gap-Closer (v. 1.2.0, Mengyang Xu *et al*. 2020) was run using these corrected ONT reads with more stringent criteria than the default parameters by requiring at least two reads to bridge a gap. We then performed a last step of high-quality short read correction of the hybrid scaffolds with Hapo-G (v 1.3, Aury and Istace 2021).

Purge Haplotigs (v. 1.1.1, Roach *et al*. 2018) was then used again to detect some potential false duplications in the hybrid scaffolds.

### Building chromosome scale assembly and structure validation

We used the RagTag tool (Alonge *et al*. 2019) with the pearl millet reference genome as a guide (Varshney *et al*. 2017, GCA_002174835.1) in order to regroup the hybrid scaffolds and construct the seven chromosomes. We launched RagTag (v. 2.1.0, Alonge *et al*. 2019) with the default parameters in order to obtain grouping, location, and orientation confidence scores for each scaffold.

However, due to potential assembly errors in the genome used as a guide, we applied more stringent criteria than the default parameters and we only kept scaffolds with a grouping confidence score above 0.7. An in-depth study, along with manual curation, was performed for one very large scaffold of 68 Mb with a grouping confidence score under 0.7. This scaffold displayed regions of tens of Mb in length aligned to two chromosomes and was identified as chimeric. It was manually cut in conformity with the alignments performed with minimap2 (v. 2.4, Li H. 2018) and visualized with D-genies (v. 1.4, Cabanettes F. and Klopp C. 2018) interactive dot plots on the two chromosomes.

In addition, we performed visual controls to check the position and the orientation of the scaffolds within each chromosome. We aligned the new chromosomes constructed with RagTag with the optical maps of another inbred genotype PMiGAP257/IP-4927 which served as a control. Optical map alignments to the new chromosomes were performed with the *runCharacterize.py* script of Bionano Solve using RefAligner and the default parameters (v. 3.3, Shelton *et al*. 2015, Yuan *et al*. 2020) and were visualized with Bionano Access (v. 3.7, Yuan *et al*. 2020). If necessary, we used reverse complement sequence and manually moved some scaffolds based on abnormalities observed in alignments (Supplementary Table 1).

To compare the new and previously obtained genome sequences, alignments were made between the chromosomes of the two assemblies using minimap2 (v. 2.24, Li H. 2018) and D-genies (v. 1.4, Cabanettes F. and Klopp C. 2018) in order to visualize alignments: we enabled the “hide noises” option and only plotted alignments with more than 50% identity. We also compared optical map alignments of the PMiGAP257/IP-4927 line between the new and the old assemblies (Varshney *et al*. 2017), to assess the improvement of the structure of the new assembly. We did not use optical maps of Tift 23D2B1-P1-P5 because since they were used for hybrid scaffolding, they showed perfect alignments with the new assembly.

To further assess the quality of the assembly of the repeat space, we calculated the long terminal repeat (LTR) assembly index (LAI) (Ou *et al.* 2018, https://github.com/oushujun/LTR_retriever, 2b702b6). We first used LTR_FINDER_parallel (v. 1.0.5, Xu, Z, Wang H 2007) and the ltr_harvest (Ellinghaus *et al*. 2008) command of genometools (v. 1.5.9, Gremme *et al*. 2013) to identify LTR elements in the assemblies. We then used LTR_retriever (v. 2.8.7, Ou and Jiang 2018) to compute the raw LAI for the old and new genome assemblies. Merqury (v. 1.3, Rhie *et al.* 2020) was used to estimate the base accuracy.

### Transposable element detection, gene completeness estimation and annotation, and centromere localization

We generated a de novo transposable elements (TEs) library from the new pearl millet assembly with RepeatModeler2 (v. 2.0.1, options—engine NCBI, Flynn *et al*. 2020). TEs were then annotated on the new assembly using RepeatMasker (v. 4.1.2, Tarailo-Graovac and Chen 2009) with the de novo TEs library. The gene completeness of both the new and the old pearl millet assemblies was estimated with BUSCO (v. 5.4.3, Manni *et al*. 2021) and the Poales dataset (odb10) composed of 4,896 genes.

Annotation of the new genome was performed with Liftoff (v 1.6.3, Shumate and Salzberg 2020) using the annotation files of the Tift 23D2B1-P1-P5 reference genome available at http://dx.doi.org/10.5524/100192 (Varshney *et al*. 2017). The genes were aligned to the new assembly with minimap2 (v2.24, Li H. 2018) and were considered correctly mapped if a minimum of 50% of the genes were aligned to the new assembly and with a sequence identity higher than 50% (-s 0.5 -a 0.5 parameters). We also enabled annotation of gene copies using a minimum identity threshold of 95% (-copies -sc 0.95 parameters). We localized the centromeric regions of the chromosomes with a satellite sequence of 137 bp specific to the pearl millet centromere (GenBank accession: Z23007.1, Kamm *et al*. 1994). We used BLAST (v. 2.9.0+, Altschul *et al*. 1990) to align and determine the positions of the centromere-specific sequence on the chromosomes of the new assembly. We only kept alignments longer than 100 bases with shared identities higher than 80%. We also aligned this satellite sequence to the hybrid scaffolds in order to further validate their orientation and positions during the building of the chromosome scale assembly.

## Results and discussion

### Optical map assembly and comparison of Tift 23D2B1-P1-P5 with the old pearl millet reference genome

A total of 1,806 Gb of data were generated for the Tift 23D2B1-P1-P5 genotype. After excluding molecules shorter than 150 kb and with fewer than 9 labels, a total of 574 Gb of data remained with an N50 of 219 kb corresponding to 383X coverage of the estimated size of the pearl millet genome. Assembly of the filtered molecules led to 164 optical maps with a total length of 1.99 Gb and a length N50 of 44.8 Mb.

A total of 90 optical maps were aligned to the old reference genome, representing a total size of 1.94 Gb with a N50 of 45.2 Mb. The remaining 74 unaligned optical maps have a N50 20 times shorter with 2.2 Mb and represented 52 Mb.

The correlation between the cumulative lengths of the optical maps assigned to each chromosome and the chromosome sizes of the old reference genome was marginally significant (Pearson correlation coefficient *r* = 0.54, *P*-value = 0.059). Chromosome 7 of the old reference genome was indeed an outlier as optical maps aligned to this chromosome were 128 Mb larger than expected (Supplementary Fig. 2). When removing chromosome 7, the correlation for the six other chromosomes was high and significant (Pearson correlation coefficient *r* = 0.90, *P*-value = 0.015).

Optical map alignments can help to identify misassembly (Yuan *et al*. 2020). We highlighted several cases of misalignments between the Tift 23D2B1-P1-P5 optical maps and the chromosomes of the old reference genome (Supplementary Figs. 3), suggesting some potential assembly errors. These misalignments are especially observed around the centromeric regions (Supplementary Table 2), as expected due to the difficulties in assembling them with short reads (Belser *et al*. 2018).

Concerning the PMiGAP257/IP-4927 genotype, a total of 2,586 Gb of data were generated. After excluding molecules shorter than 150 kb and with fewer than nine labels, a total of 685 Gb of data remained with an N50 of 213 kb corresponding to 403X coverage of the estimated pearl millet genome. Assembly of the filtered molecules led to 346 assembled optical maps with a total length of 2.48 Gb and a N50 length of 25.2 Mb.

### Long reads ONT assembly and hybrid scaffolding

A total of 6,261,759 ONT reads from the Tift 23D2B1-P1-P5 genotype were generated with a cumulative size of 108 Gb, corresponding to a mean depth of 60X. For the assembly, we only kept reads with a quality score higher than 10 and larger than 5 kb. A total of 2,640,214 long reads remained, with a read length N50 of 25.2 kb and a mean size of 21.8 kb after quality filtering. The total sequence data amount used for the assembly was 57.6 Gb, corresponding to a mean depth of 32X for this inbred genotyped.

Assembly with Flye and polishing led to 3,641 ONT contigs with a N50 of 1.2 Mb. The N50 length of the contigs is 67 times longer than the contigs N50 obtained from the previous Tift 23D2B1-P1-P5 genome (Table 1, Varshney *et al*. 2017).

**Table 1.** Statistics of the pearl millet Tift 23D2B1-P1-P5 old reference genome and of the new assembly.

	Old reference genome	New assembly
**Total assembly**		
Total length	1.82 Gb	1.85 Gb
GC content	47.9%	49.5%
Complete BUSCO scores	94.4%	98.4%
**Chromosomes**		
Number of chr	7	7
Total length of chr	1,564,537,551 bp	1,778,181,882 bp
Percentage of Ns	13.5%	0.3%
**Scaffolds**		
Number of scaffolds	25,241	72
Longest scaffold	4,816,714 bp	167,249,600 bp
N50 (scaffolds)	884,945 bp	85,795,566 bp
**Contigs**		
Number of contigs	175,708	3,641
Longest contig	282,901 bp	6,842,273 bp
N50 (contigs)	18,180 bp	1,209,791 bp

Hybrid scaffolding of ONT contigs using the Bionano optical maps led to 72 hybrid scaffolds with a cumulative length of 1.86 Gb. The N50 length of these scaffolds is 86 Mb, which is roughly 100 times greater than the previous assembly (Table 1, Varshney *et al*. 2017). The total length of the remaining 1,161 unplaced ONT contigs represented 55 Mb with a N50 of 68 kb. We finalized this hybrid scaffolding by bridging gaps with TGS Gap-Closer, leading to a strong decrease in N bases from 4.31 to 0.29%.

### Reference guided chromosome construction

Of the 72 hybrid scaffolds, 53 displayed a grouping confidence score above 0.7 to a single chromosome using RagTag. One scaffold showed ∼42 Mb aligned to chromosome 5 and ∼26 Mb aligned to chromosome 4 and was therefore identified as chimeric and manually split (Supplementary Table 1: Scaffold_8135). The two split scaffolds then showed high grouping confidence scores (Supplementary Table 1).

**Fig. 1.**
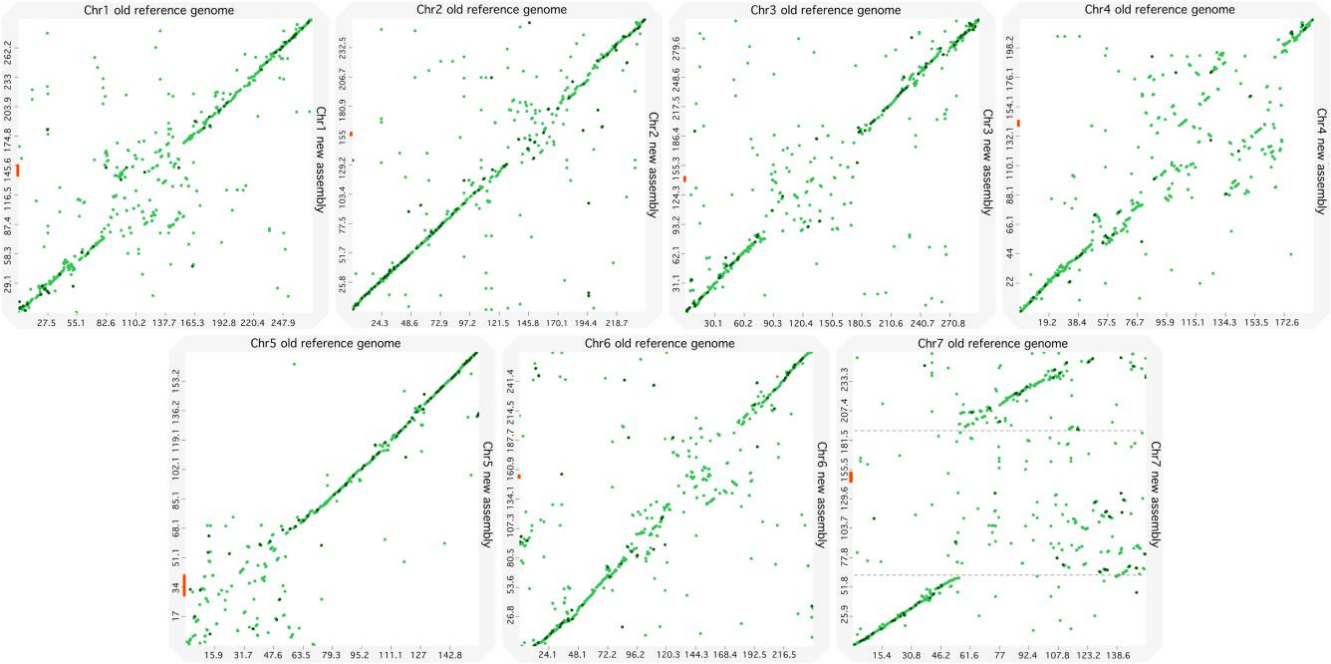
Alignments between the chromosomes of the new assembly and the chromosomes of the old Tift 23D2B1-P1-P5 reference genome. Plots of the alignments obtained with D-genies are shown between the old reference genome (Varshney *et al*. 2017) on the horizontal axis and the new assembly on the vertical axis. Chromosome lengths on the axis are given in Mbases. Alignments with identities between 50 and 75% are in light-green and in dark-green are alignments with identities between 75 and 100%. The thick lines on the vertical axis correspond to the positions of the centromeric satellite sequence on each chromosome of the new assembly. A large region of more than 100 Mb is missing on the chromosome 7 of the old reference genome: this region is marked out with dotted grey lines on the chromosome of the new assembly.

We also manually reversed the sequence of a scaffold of 88 Mb assigned to chromosome 3 with low orientation confidence score (Supplementary Table 1: Scaffold_1980). Alignments of the centromeric repetitive sequence were found both at the beginning of Scaffold_1980 and at the beginning of the following scaffold (Supplementary Table 1: Scaffold_3136), which supported the decision to reverse Scaffold_1980. Orientation of this large scaffold was confirmed when comparing the new assembly both with the optical maps of the control line PMiGAP257/IP-4927 and with the chromosome 3 of the previous reference genome (Fig. 1).

Two other large scaffolds of 147 and 105 Mb also displayed good but below 0.7 grouping confidence scores to chromosome 7 (Supplementary Table 1: Scaffold_1301 and Scaffold_2567). These two large scaffolds led to a new chromosome 7 around 105 Mb larger than chromosome 7 of the reference genome (Varshney *et al*. 2017), in accordance with the inference made previously with the optical maps (Supplementary Fig. 2). In addition, centromere-specific sequence repeats were identified at the extremities of these two large scaffolds positioned one after another and further supported their positions and their orientations. Because we also discarded the possibility of major duplications in the new assembly using Purge Haplotigs analysis (Supplementary Fig. 4), we hypothesized that the centromeric region of the chromosome 7 was previously not well assembled in the reference genome and assigned these two scaffolds to the new chromosome 7. This was validated by subsequent analyses presented in the next section.

The total length of the new final assembly was 1.85 Gb and the cumulative size of the chromosomes was 1.78 Gb. This is very close to the pearl millet estimated genome size (1.76 Gb, Varshney *et al*. 2017). We assembled 96% of the genome on chromosomes compared to 87% in the previous Tift 23D2B1-P1-P5 assembly (Varshney *et al*. 2017) which corresponds to more than 200 additional Mb at the chromosome-level assembly. Chromosomes also displayed very low Ns content (0.29%) compared to the chromosomes of the previous assembly (above 13%).

The QV score obtained for the genic space was high 30.6, but lower when we considered also the repetitive space (QV = 23.6). Illumina short reads (Varshney *et al*. 2017) and Nanopore reads do not come from the exact same individuals, and some small variations (<1%) might exist between individuals of the same inbred line (Mascher *et al*. 2014). Polishing repetitive regions is still difficult (Belser *et al*. 2021) and certainly contributes to lowering the QV score in the repetitive space.

### Gene completeness and structure accuracy of the assembly

The percentage of complete BUSCO genes of the Poales database found in the new assembly was 98.4%, an increase of 4% from the previous reference genome (Table 1). Only 3.3% of the BUSCO genes were duplicated genes in the new assembly. This figure is in accordance with that expected (Guan *et al*. 2020). The percentage of interspersed repeats found on the new assembly was 82.1% including 64.1% of LTR elements, a percentage also in accordance with previous study (Varshney *et al*. 2017).

Concerning the 38,579 gene models from the Tift 23D2B1-P1-P5 pearl millet reference genome (Varshney *et al*. 2017), 37,814 sequences (98.0%) were mapped at least once to the new assembly with a mean coverage and a mean identity of 97.5 and 96.2%, respectively. A total of 36,898 genes (95.6%) were found on the seven new chromosomes. This improved the number of genes found on chromosomes by 1,107 compared to the previous Tift 23D2B1-P1-P5 reference. Both the BUSCO scores and mapping of genes to the new assembly revealed enhanced gene completeness in the new chromosomal sequences.

A large region of more than 100 Mb was added to the chromosome 7 of the new assembly. An excess of genes annotated on the unplaced scaffolds of the previous reference genome was mapped to this new chromosome 7: of the 2,342 genes originating from the unplaced scaffolds of the previous assembly and mapped to the new chromosomes, a total of 1,101 genes (47%) were found on the new chromosome 7. This observation added weight to our longer assembly for chromosome 7.

The alignments of our new assembly with the previous Tift 23D2B1-P1-P5 reference genome showed overall good matches all along the chromosomes, particularly at the extremities (Fig. 1). The regions around the centromeres (Supplementary Table 2) showed the strongest divergence in alignments (Fig. 1), a pattern previously observed and expected in comparisons between long read and short read assemblies (Belser *et al*. 2018).

Optical map alignments with the PMiGAP257/IP-4927 line enabled us to validate the order and the orientation of the scaffolds within the new manually curated chromosomes (Supplementary Fig. 5). In addition, PMiGAP257/IP-4927 optical map alignments with both the new and the previous assemblies helped us to assess the improvement of the structure and of the continuity of the new chromosomes. Alignments of these optical maps to the seven new chromosomes showed much better overall continuity compared to the previous Tift 23D2B1-P1-P5 reference genome (Supplementary Fig. 5). The better alignments are particularly noticeable in the centromeric regions (Supplementary Fig. 5). A better continuity in the assembly of the repetitive sequences of the new genome was also observed with a three-fold increase of the LAI score between the new assembly (LAI = 18.5) and the old genome (LAI = 6.5). This LAI score is closed to the threshold value of 20 attributed to gold quality assembly (Ou *et al*. 2018).

### Conclusion

We present here an assembly obtained with both Oxford Nanopore long reads and Bionano Genomics optical maps for the pearl millet Tift 23D2B1-P1-P5 cultivar genotype. This assembly displays improvement compared to the previous pearl millet reference genome (Varshney *et al*. 2017), in terms of both continuity and gene completeness. Obtaining high-quality references is important to be able to study genomic diversity and structural variants in a species. This new version will thus help us to better study structural variants within pearl millet populations.

## Supplementary Material

jkad051_Supplementary_Data

## Data Availability

The Tift 23D2B1-P1-P5 (BioSample identifier: SAMN04124419) pearl millet reference assembly (Varshney *et al*. 2017) is available both in the NCBI (ASM217483v1) and through the European Nucleotide Archive (GCA_002174835.1). Raw Illumina short reads from the Tift 23D2B1-P1-P5 genotype used for assemblies and ONT long reads polishing are accessible in NCBI with SRA accession SRP063925 (list of SRR identifiers used: SRR2489264-SRR2489273). Transfer annotation to the new assembly was performed using the genome annotation file pearl_millet_gff.gz available at http://dx.doi.org/10.5524/100192. The new chromosome-level assembly of the Tift 23D2B1-P1-P5 genotype (the 7 chromosomes are available under the accession GCA_947561735.1, https://www.ebi.ac.uk/ena/browser/view/GCA_947561735.1, and the chromosomes plus the unplaced sequences under the accession GCA_947561735.3) and data used for this study including the ONT long reads (run accession: ERR10627707) and the Bionano optical maps (analysis accessions: ERZ14864807 for PMiGAP257/IP-4927 and ERZ14865266 for Tift 23D2B1-P1-P5) have been deposited in the European Nucleotide Archive under the study accession PRJEB57746. The gff file is also available under ENA, with the accession ERZ15184682. Supplemental material available at G3 online.

## References

[jkad051-B1] Alonge M , Soyk S, Ramakrishnan S, Wang X, Goodwin S, Sedlazeck FJ, Lippman ZB, Schatz MC. RaGOO: fast and accurate reference-guided scaffolding of draft genomes. Genome Biol. 2019;20:224. doi:10.1186/s13059-019-1829-6. 31661016 PMC6816165

[jkad051-B2] Altschul SF , Gish W, Miller W, Myers EW, Lipman DJ. Basic local alignment search tool. J Mol Biol. 1990;215(3):403–410. doi:10.1016/S0022-2836(05)80360-2. 2231712

[jkad051-B3] Aury JM , Engelen S, Istace B, Monat C, Lasserre-Zuber P, Belser C, Cruaud C, Rimbert H, Leroy P, Arribat S, et al Long-read and chromosome-scale assembly of the hexaploid wheat genome achieves high resolution for research and breeding. Gigascience. 2022;11:giac034. doi:10.1093/gigascience/giac034. 35482491 PMC9049114

[jkad051-B4] Aury JM , Istace B. Hapo-G, haplotype-aware polishing of genome assemblies with accurate reads. NAR Genom Bioinform. 2021;3(2):879. doi:10.1093/nargab/lqab034. PMC8092372 33987534

[jkad051-B5] Belser C , Baurens FC, Noel B, Martin G, Cruaud C, Istace B, Yahiaoui N, Labadie K, Hřibová E, Doležel J, et al Telomere-to-telomere gapless chromosomes of banana using nanopore sequencing. Commun Biol. 2021;4(1):1047. doi:10.1038/s42003-021-02559-3. 34493830 PMC8423783

[jkad051-B6] Belser C , Istace B, Denis E, Dubarry M, Baurens FC, Falentin C, Genete M, Berrabah W, Chèvre AM, Delourme R, et al Chromosome-scale assemblies of plant genomes using nanopore long reads and optical maps. Nat Plants. 2018;4(11):879–887. doi:10.1038/s41477-018-0289-4. 30390080

[jkad051-B7] Cabanettes F , Klopp C. D-GENIES: dot plot large genomes in an interactive, efficient and simple way. PeerJ. 2018;6:e4958. doi:10.7717/peerj.4958. 29888139 PMC5991294

[jkad051-B8] Danecek P , Bonfield JK, Liddle J, Marshall J, Ohan V, Pollard MO, Whitwham A, Keane T, McCarthy SA, Davies RM, et al Twelve years of SAMtools and BCFtools. Gigascience. 2021;10(2):giab008. doi:10.1093/gigascience/giab008. 33590861 PMC7931819

[jkad051-B9] De Coster W , D'Hert S, Schultz DT, Cruts M, Van Broeckhoven C. Nanopack: visualizing and processing long-read sequencing data. Bioinformatics. 2018;34(15):2666–2669. doi:10.1093/bioinformatics/bty149. 29547981 PMC6061794

[jkad051-B10] Ellinghaus D , Kurtz S, Willhoeft U. LTRharvest, an efficient and flexible software for de novo detection of LTR retrotransposons. BMC Bioinformatics. 2008;9:18. doi:10.1186/1471-2105-9-18. 18194517 PMC2253517

[jkad051-B11] Flynn JM , Hubley R, Goubert C, Rosen J, Clark AG, Feschotte C, Smit AF. Repeatmodeler2 for automated genomic discovery of transposable element families. Proc Natl Acad Sci U S A. 2020;117(17):9451–9457. doi:10.1073/pnas.1921046117. 32300014 PMC7196820

[jkad051-B12] Gremme G , Steinbiss S, Kurtz S. Genometools: a comprehensive software library for efficient processing of structured genome annotations. IEEE/ACM Trans Comput Biol Bioinform. 2013;10(3):645–656. doi:10.1109/TCBB.2013.68. 24091398

[jkad051-B13] Guan D , McCarthy SA, Wood J, Howe K, Wang Y, Durbin R. Identifying and removing haplotypic duplication in primary genome assemblies. Bioinformatics. 2020;36(9):2896–2898. doi:10.1093/bioinformatics/btaa025. 31971576 PMC7203741

[jkad051-B14] Huang K , Rieseberg LH. Frequency, origins, and evolutionary role of chromosomal inversions in plants. Front Plant Sci. 2020;11:296. doi:10.3389/fpls.2020.00296. 32256515 PMC7093584

[jkad051-B15] Istace B , Belser C, Aury JM. BiSCoT: improving large eukaryotic genome assemblies with optical maps. PeerJ. 2020;8:e10150. doi:10.1101/674721. 33194395 PMC7649008

[jkad051-B16] Istace B , Belser C, Falentin C, Labadie K, Boideau F, Deniot G, Maillet L, Cruaud C, Bertrand L, Chèvre AM, et al Sequencing and chromosome-scale assembly of plant genomes, *Brassica rapa* as a use case. Biology (Basel). 2021;10:732. doi:10.3390/biology10080732. 34439964 PMC8389630

[jkad051-B17] Kamm A , Schmidt T, Heslop-Harrison JS. Molecular and physical organization of highly repetitive, undermethylated DNA from Pennisetum glaucum. Mol Gen Genet. 1994;244(4):420–425. doi:10.1007/BF00286694. 7521511

[jkad051-B18] Kolmogorov M , Yuan J, Lin Y, Pevzner PA. Assembly of long, error-prone reads using repeat graphs. Nat Biotechnol. 2019;37(5):540–546. doi:10.1038/s41587-019-0072-8. 30936562

[jkad051-B19] Li H . Minimap2: pairwise alignment for nucleotide sequences. Bioinformatics. 2018;34:3094–3100. doi:10.1093/bioinformatics/bty191. 29750242 PMC6137996

[jkad051-B20] Li H , Durbin R. Fast and accurate long-read alignment with Burrows-Wheeler transform. Bioinformatics. 2010;26:589–595. doi:10.1093/bioinformatics/btp698. 20080505 PMC2828108

[jkad051-B21] Manni M , Berkeley MR, Seppey M, Zdobnov EM. BUSCO: assessing genomic data quality and beyond. Curr Protoc. 2021;1(12):e323. doi:10.1002/cpz1.323. 34936221

[jkad051-B22] Mariac C , Zekraoui L, Leblanc O. High molecular weight DNA extraction from plant nuclei isolation. Protocols.io. 2019. 10.17504/protocols.io.83shyne.

[jkad051-B23] Martin M . Cutadapt removes adapter sequences from high-throughput sequencing reads. EMBnet.j. 2011;17(1):10–12. doi:10.14806/ej.17.1.200.

[jkad051-B24] Mascher M , Gerlach N, Gahrtz M, Bucher M, Scholz U, Dresselhaus T. Sequence and ionomic analysis of divergent strains of maize inbred line B73 with an altered growth phenotype. PLoS One. 2014;9(5):e96782. doi:10.1371/journal.pone.0096782. 24804793 PMC4013074

[jkad051-B25] Orjuela J , Comte A, Ravel S, Charriat F, Vi T, Sabot F, Cunnac S. CulebrONT: a streamlined long reads multi-assembler pipeline for prokaryotic and eukaryotic genomes. Peer Community J. 2022;2:E46. doi:10.24072/pcjournal.153.

[jkad051-B26] Ou S , Chen J, Jiang N. Assessing genome assembly quality using the LTR Assembly Index (LAI). Nucleic Acids Res. 2018;46(21):e126. doi:10.1093/nar/gky730. 30107434 PMC6265445

[jkad051-B27] Ou S , Jiang N. LTR_Retriever: a highly accurate and sensitive program for identification of long terminal repeat retrotransposons. Plant Physiol. 2018;176(2):1410–1422. doi:10.1104/pp.17.01310. 29233850 PMC5813529

[jkad051-B28] Rhie A , Walenz BP, Koren S, Phillippy AM. Merqury: reference-free quality, completeness, and phasing assessment for genome assemblies. Genome Biol. 2020;21(1):133. doi:10.1186/s13059-020-02134-9. 32928274 PMC7488777

[jkad051-B29] Roach MJ , Schmidt SA, Borneman AR. Purge Haplotigs: allelic contig reassignment for third-gen diploid genome assemblies. BMC Bioinformatics. 2018;19(1):460. doi:10.1186/s12859-018-2485-7. 30497373 PMC6267036

[jkad051-B30] Shelton JM , Coleman MC, Herndon N, et al Tools and pipelines for BioNano data: molecule assembly pipeline and FASTA super scaffolding tool. BMC Genomics. 2015;16:734. doi:10.1186/s12864-015-1911-8. 26416786 PMC4587741

[jkad051-B31] Shumate A , Salzberg SL. Liftoff: accurate mapping of gene annotations. Bioinformatics. 2020;37(12):1639–1643. doi:10.1093/bioinformatics/btaa1016. PMC8289374 33320174

[jkad051-B32] Tarailo-Graovac M , Chen N. Using RepeatMasker to identify repetitive elements in genomic sequences. Curr Protoc Bioinformatics. 2009;Chapter 4:4.10.1–4.10.14. doi:10.1002/0471250953.bi0410s25. 19274634

[jkad051-B33] Varshney RK , Shi C, Thudi M, Mariac C, Wallace J, Qi P, Zhang H, Zhao Y, Wang X, Rathore A, et al Pearl millet genome sequence provides a resource to improve agronomic traits in arid environments. Nat Biotechnol. 2017;35(10):969–976. 10.1038/nbt.3943. 28922347 PMC6871012

[jkad051-B34] Vaser R , Sović I, Nagarajan N, Šikić M. Fast and accurate de novo genome assembly from long uncorrected reads. Genome Res. 2017;27(5):737–746. https://doi:10.1101/gr.214270.116. 28100585 10.1101/gr.214270.116 PMC5411768

[jkad051-B35] Vasimuddin M , Misra S, Li H, Aluru S. 2019. Efficient architecture-aware acceleration of BWA-MEM for multicore systems. In: IEEE International Parallel and Distributed Processing Symposium (IPDPS). Rio de Janeiro (Brazil): IEEE. p. 314–324.

[jkad051-B36] Wellenreuther M , Bernatchez L. Eco-evolutionary genomics of chromosomal inversions. Trends Ecol Evol. 2018;33(6):427–440. doi:10.1016/j.tree.2018.04.002. 29731154

[jkad051-B37] Wickham H . ggplot2: Elegant Graphics for Data Analysis. New York: Springer-Verlag; 2016.

[jkad051-B38] Xu M , Guo L, Gu S, Wang O, Zhang R, Peters BA, Fan G, Liu X, Xu X, Deng L, et al TGS-GapCloser: a fast and accurate gap closer for large genomes with low coverage of error-prone long reads. GigaScience. 2020;9(9):giaa094. doi:10.1093/gigascience/giaa094. 32893860 PMC7476103

[jkad051-B39] Xu Z , Wang H. LTR_FINDER: an efficient tool for the prediction of full-length LTR retrotransposons. Nucleic Acids Res. 2007;35(Web Server issue):W265–W268. doi:10.1093/nar/gkm286. 17485477 PMC1933203

[jkad051-B40] Yuan Y , Chung CY, Chan TF. Advances in optical mapping for genomic research. Comput Struct Biotechnol J. 2020;18:2051–2062. doi:10.1016/j.csbj.2020.07.018. 32802277 PMC7419273

