# Supplementary material for: An improved assembly of the pearl millet reference genome using Oxford Nanopore long reads and optical mapping
Source: G3 (Bethesda). 2023 Mar 9;13(5):jkad051. doi: 10.1093/g3journal/jkad051 (PMC10151396; doi:10.1093/g3journal/jkad051)
Supplement: jkad051_Supplementary_Data [file jkad051_supplementary_data.zip › Table_S2_G3-2022-403975.pdf]

Table S2 Positions of the centromeric specific sequence on the chromosomes of the new assembly

	Positions of alignments	Number of alignments
chr1	134.1 - 147.8 Mb	93/93 (100%)
chr2	154.9 - 156.9 Mb	47/54 (87%)
chr3	138.8 - 143.7 Mb	1981/1981 (100%)
chr4	139.5 - 144.4 Mb	57/57 (100%)
chr5	29.2 - 44.9 Mb	208/208 (100%)
chr6	154.0 - 155.2 Mb	410/411 (99%)
chr7	144.2 - 153.9 Mb	102/102 (100%)

The 137 bp centromere specific sequence (Kamm et al. 1994) was aligned to each chromosome of the new assembly using blast (v. 2.9.0+, Altschul et at 1990). We only kept alignments longer than 100 bases and with an identity higher than 80%. Each line of the table has to be ridden as follows: for chromosome 1, 100% of the filtered alignments were found between 134.1 and 147.8 Mb.
