# Supplementary material for: An improved assembly of the pearl millet reference genome using Oxford Nanopore long reads and optical mapping
Source: G3 (Bethesda). 2023 Mar 9;13(5):jkad051. doi: 10.1093/g3journal/jkad051 (PMC10151396; doi:10.1093/g3journal/jkad051)

Figure S3 (to be continued)

Chromosome 1

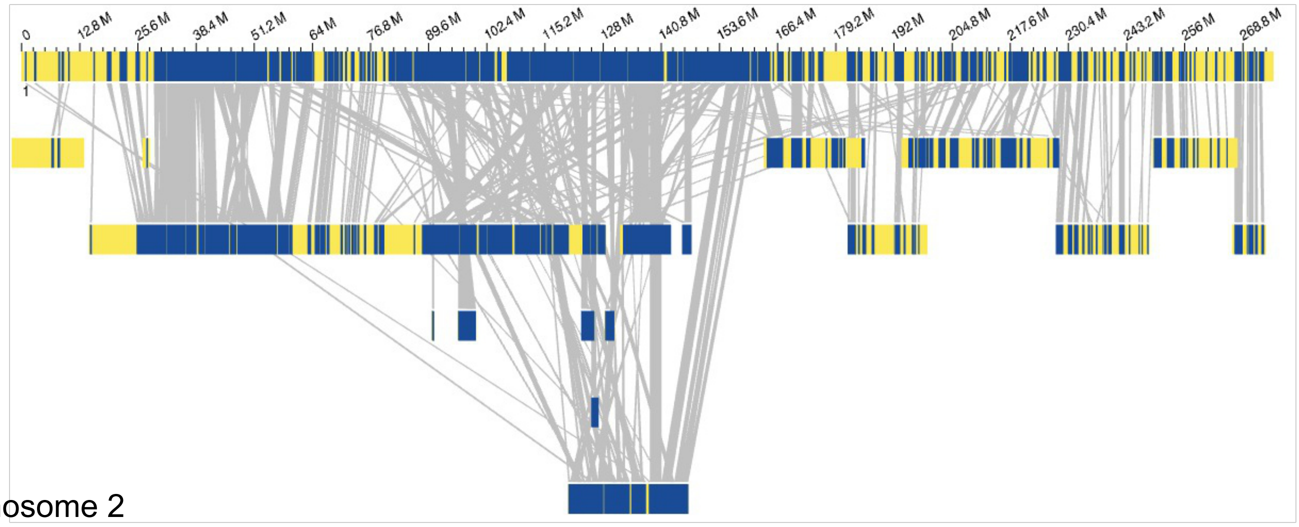

Chromosome 2

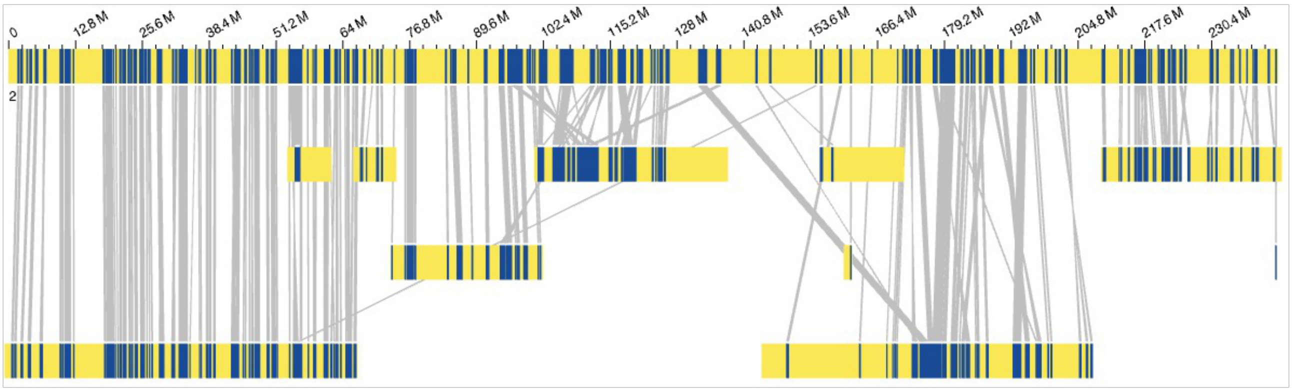

Chromosome 3

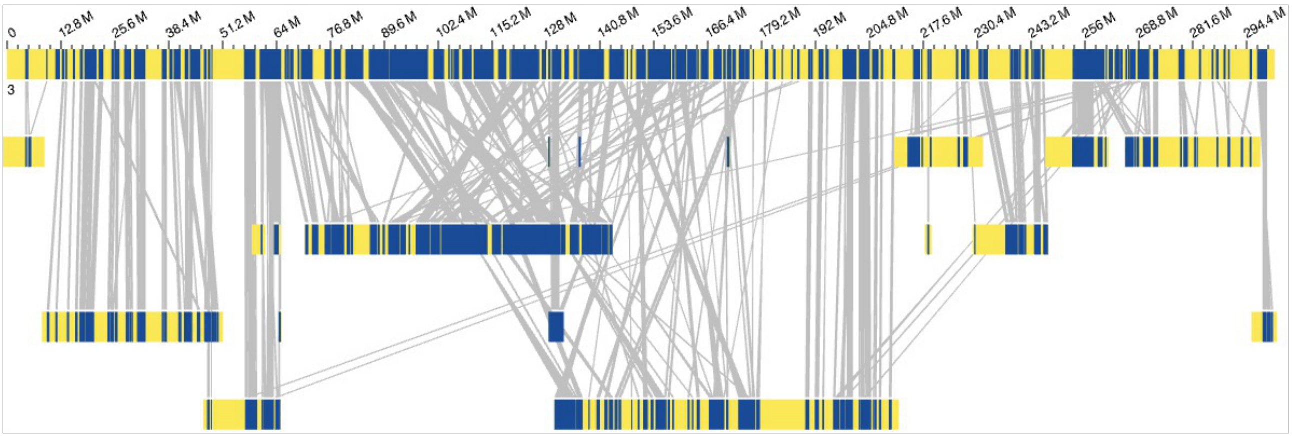

Chromosome 4

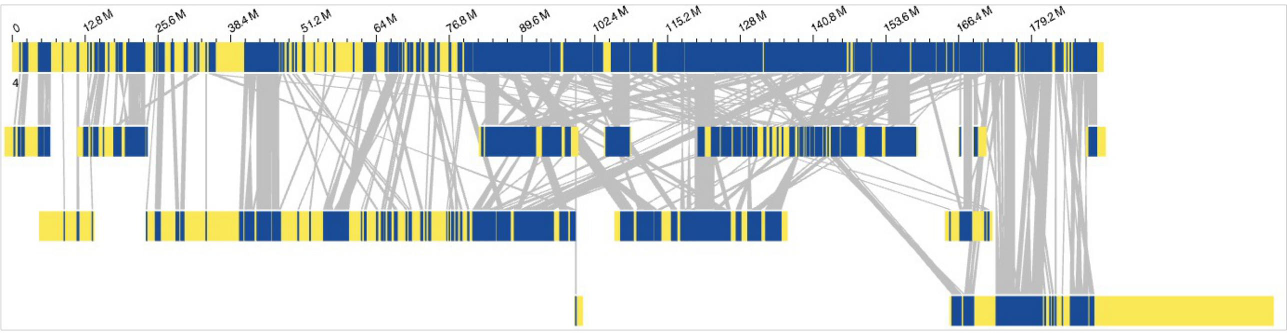

Figure S3 (continued)

Chromosome 5

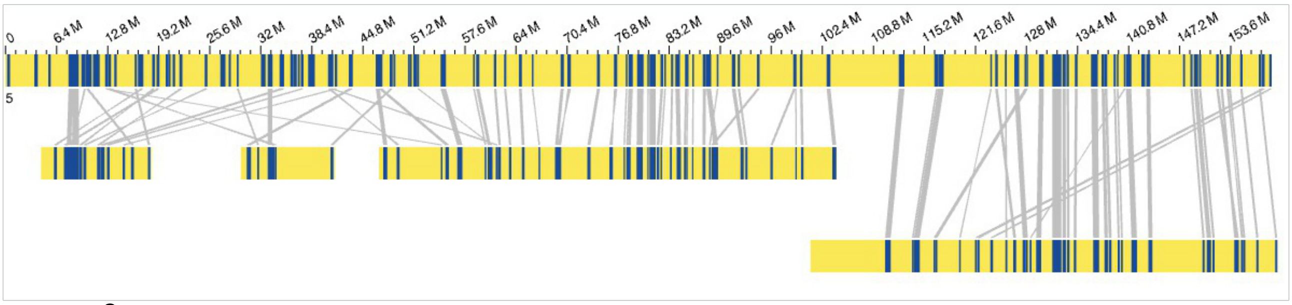

Chromosome 6

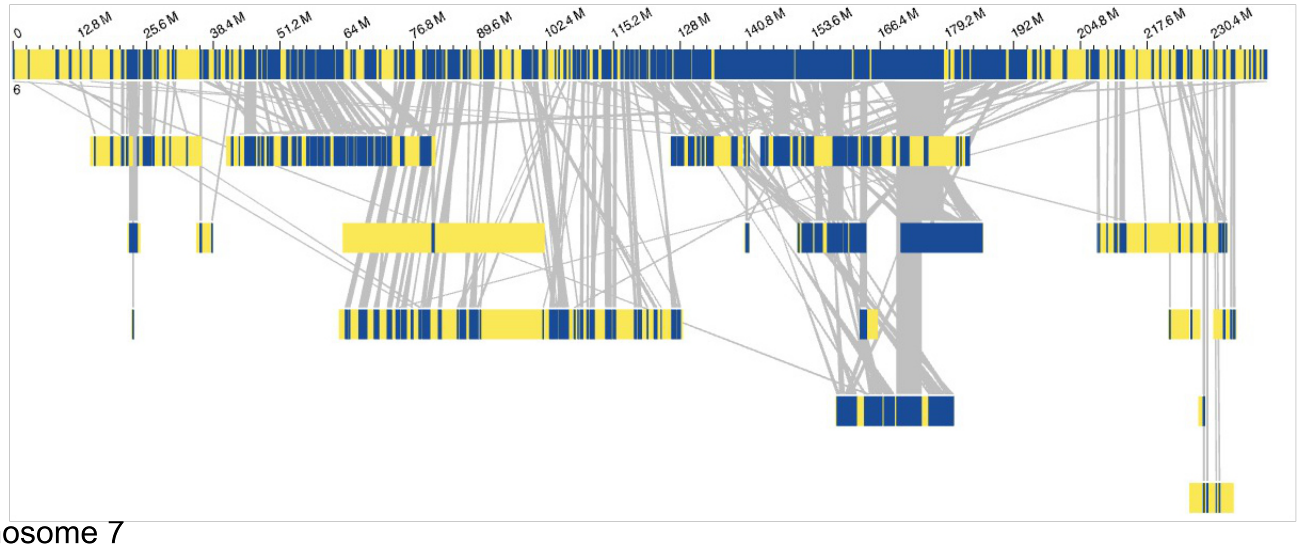

Chromosome 7

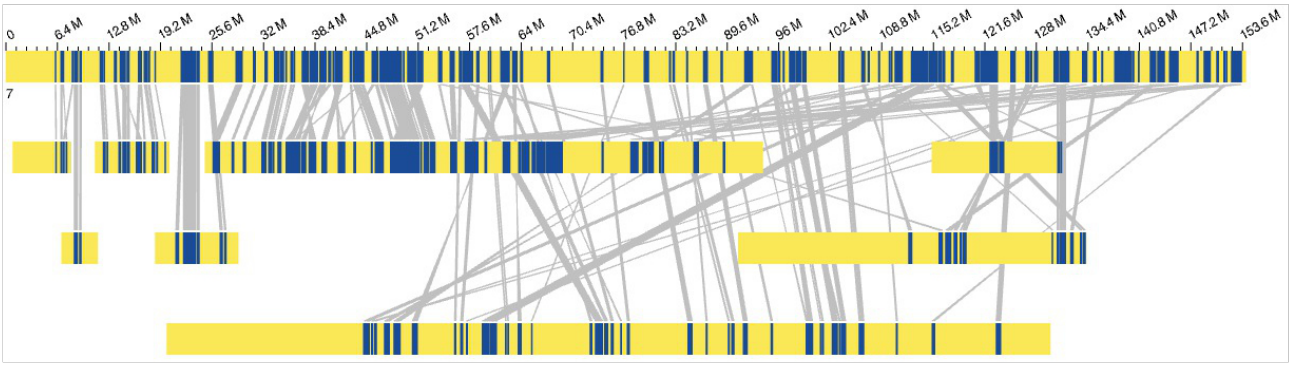

Supplement: jkad051_Supplementary_Data [file jkad051_supplementary_data.zip › Figure_S3_G3-2022-403975.pdf]
