# Supplementary material for: An improved assembly of the pearl millet reference genome using Oxford Nanopore long reads and optical mapping
Source: G3 (Bethesda). 2023 Mar 9;13(5):jkad051. doi: 10.1093/g3journal/jkad051 (PMC10151396; doi:10.1093/g3journal/jkad051)

Figure S5 (to be continued)

chromosome 1

chromosome 2

chromosome 3

chromosome 4

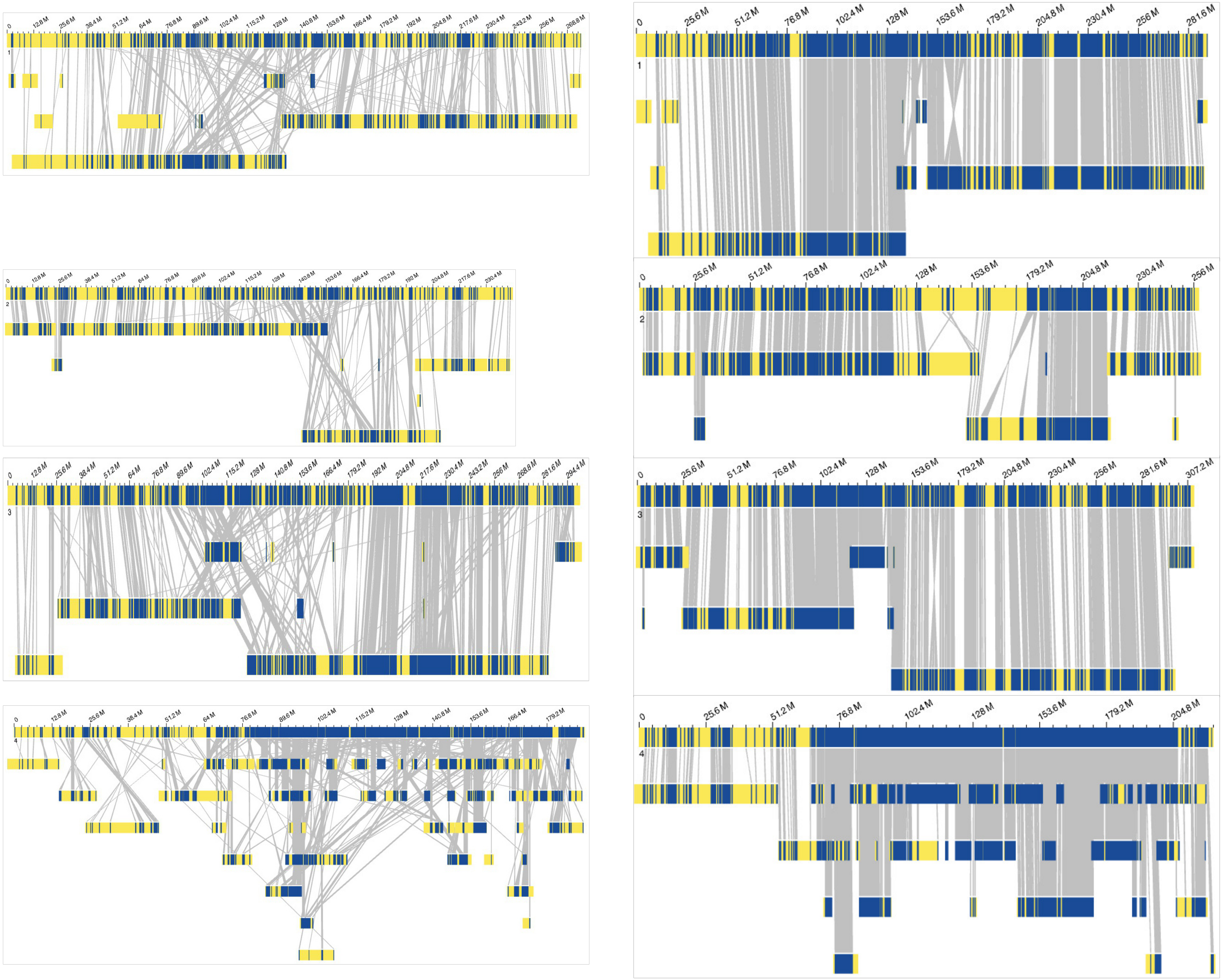

Figure S5 (continued)

chromosome 5

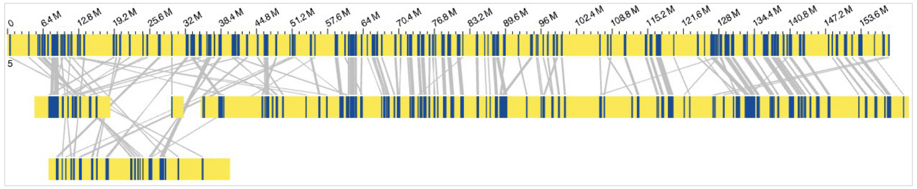

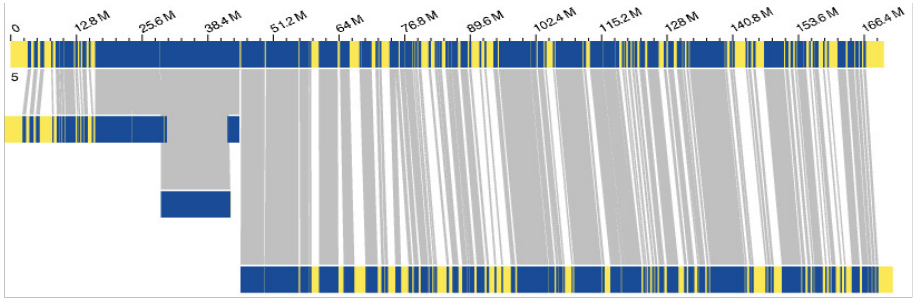

chromosome 6

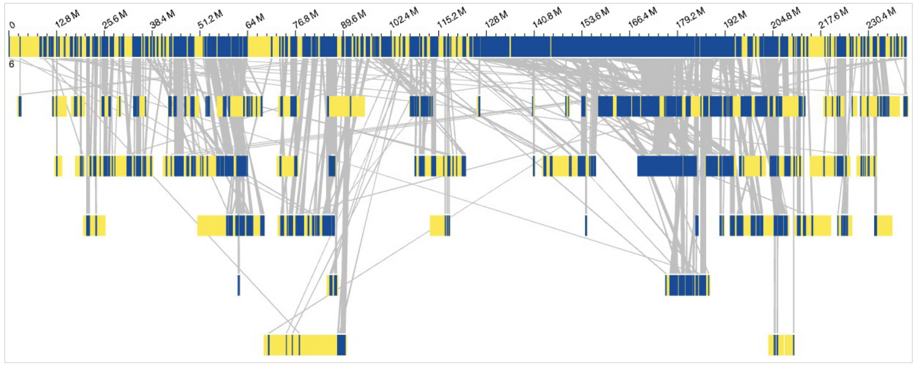

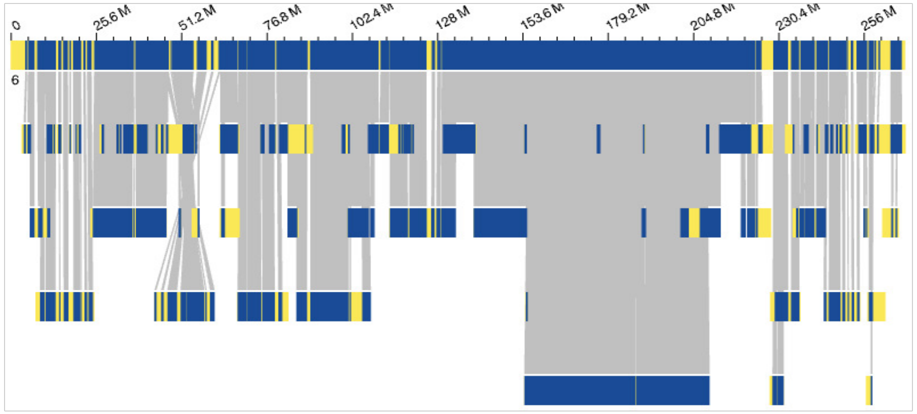

chromosome 7

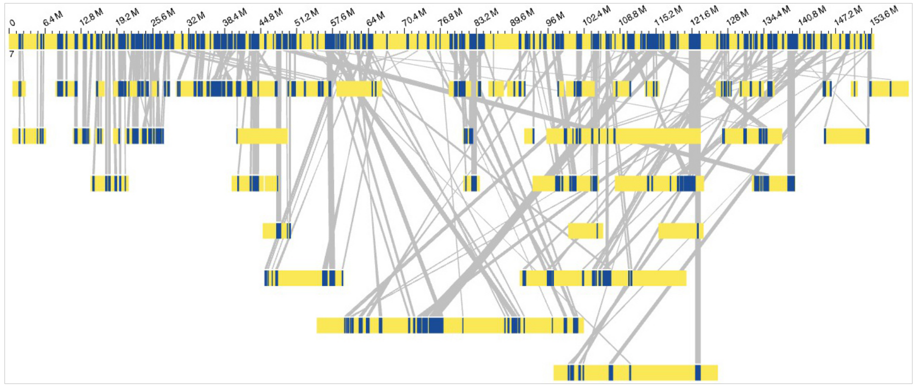

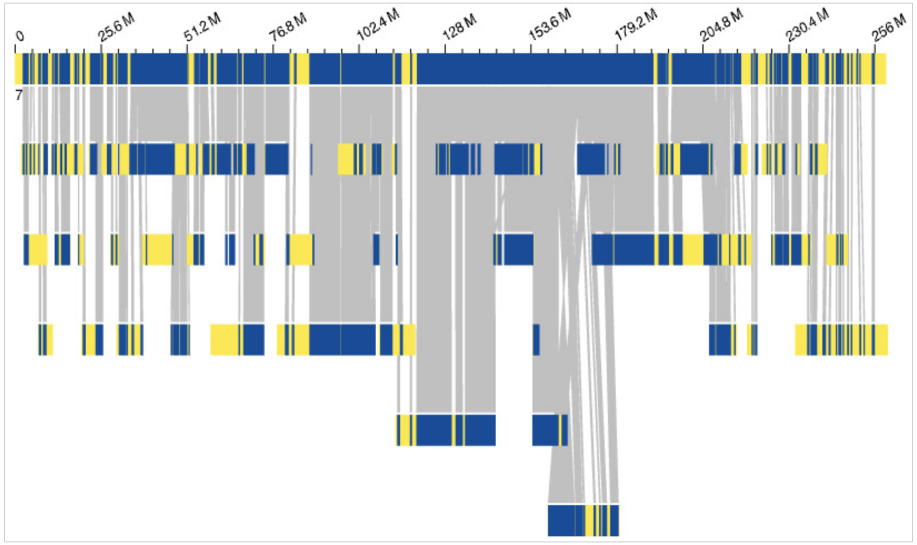

Supplement: jkad051_Supplementary_Data [file jkad051_supplementary_data.zip › Figure_S5_G3-2022-403975.pdf]
