# Supplementary material for: An improved assembly of the pearl millet reference genome using Oxford Nanopore long reads and optical mapping
Source: G3 (Bethesda). 2023 Mar 9;13(5):jkad051. doi: 10.1093/g3journal/jkad051 (PMC10151396; doi:10.1093/g3journal/jkad051)
Supplement: jkad051_Supplementary_Data [file jkad051_supplementary_data.zip › Supplemental_Figure_Legend_G3-2022-403975.docx]

**Figure S1** Pipeline of the genome assembly combining long reads and optical mapping.

Assembly of the ONT long reads was performed with the assembler Flye. Two rounds of Racon and Medaka were used to polish and correct the ONT contigs using the long reads. The ONT contigs were polished with high quality Illumina short reads using Hapo-G. *De novo* assembly of the Bionano molecules was performed using Bionano Solve pipeline. Hybrid scaffolding of the ONT contigs was performed using the Bionano assembled optical maps with Bionano Solve. ONT contigs not aligned to an optical map were placed in the chrUN. We used BiSCoT in order to remove artefactual duplications from the hybrid scaffolds. TGS Gap-Closer was used to perform gap filling and reduce the total number of Ns in the hybrid scaffolds. A last step of high quality short reads correction with Hapo-G was performed. Chromosomes were finally builded using RagTag and the pearl millet Tift 23D2B1-P1-P5 reference genome (Varshney et al. 2017) as a guide. Manual curations were performed based on RagTag confidence scores and hybrid scaffolds with grouping confidence scores below 0.7 were added to the chrUN.

**Figure S2** Correlation between the chromosomes size estimated using optical maps and the chromosomes length of the old reference genome.

The correlation is marginally significant (Pearson correlation coefficient r=0.736, p-value=0.059). The size of chromosome 7 of the old reference genome (Varshney et al. 2017) appeared underestimated by roughly 128 Mb.

**Figure S3** Comparison between the chromosomes of the old pearl millet reference genome and the optical maps of the same genotype Tift 23D2B1-P1-P5

We show optical map alignments to each chromosome of the old reference genome (Varshney et al. 2017) using Bionano Access. Dark blue color corresponds to regions where labels are aligned between the optical maps and the reference, and gray lines join the aligned labels between them. Yellow color represents regions without label matches. Several cases of crossing lines between the old reference genome and the optical maps are shown. This pattern suggests discontinuity between the order of the contigs and scaffolds in the assembly of the old reference genome and the optical maps.

**Figure S4** Read depth histogram obtained with Purge Haplotigs

The histogram represents the total number of bases of the assemblies (on the vertical axis) with a given read-depth (on the horizontal axis). A corresponds to the ONT contigs and B to the hybrid scaffolds before the building of chromosomes. No evidence of duplication is shown in the plot. The pic at a read-depth equal to 0 corresponds to Ns regions between ONT contigs positioned on the hybrid scaffolds.

**Figure S5** Comparison of the alignments between the PMiGAP257/IP-4927 optical maps and the chromosomes of both the old and the new assembly

Optical maps from the PMiGAP257/IP-4927 line were aligned to both the new and the old reference genomes with Bionano Solve: the chromosomes on the left correspond to the old reference genome (Varshney et al. 2017) and the chromosomes on the right correspond the new assembly. Dark blue color corresponds to regions where labels are aligned between the optical maps and the genomes, and gray lines join the aligned labels between them. Yellow color represents regions without label matches. Overall, the chromosomes of the new assembly on the right showed less crossing lines with the optical maps of PMiGAP257/IP-4927 line, a signature of better continuity of the order of the contigs and scaffolds in the new assembly.
