# Supplementary material for: An improved assembly of the pearl millet reference genome using Oxford Nanopore long reads and optical mapping
Source: G3 (Bethesda). 2023 Mar 9;13(5):jkad051. doi: 10.1093/g3journal/jkad051 (PMC10151396; doi:10.1093/g3journal/jkad051)
Supplement: jkad051_Supplementary_Data [file jkad051_supplementary_data.zip › Table_S1_G3-2022-403975.pdf]

Table S1 (to be continued) Grouping, location and orientation confidence scores obtained with RagTag and assignation of the scaffolds to chromosomes 1, 2 and 3

scaffolds	positions	Length (Mb)	Grouping confidence	Location confidence	Orientatin confidence	Assignation	Orientation	Manual curation	Commentary
Scaffold_100110	1	0.23	1.00	1.00	1.00	**chr1**	+		
Scaffold_100122	226189	0.24	0.89	0.01	1.00	**chr1**	-		
Scaffold_334	466543	0.38	0.57	0.00	0.48	**chrUN**			
Scaffold_1755	848567	0.28	0.36	1.00	1.00	**chrUN**			
Scaffold_684	1125736	135.22	0.89	0.43	0.67	**chr1**	+		
Scaffold_100099	136349156	0.34	0.82	0.00	1.00	**chr1**	-		
Scaffold_3432	136691461	3.88	0.99	0.76	0.84	**chr1**	+		
Scaffold_100068	140568769	2.74	0.97	0.10	0.75	**chr1**	+	reversed	Optical maps alignments
Scaffold_100077	143312877	1.21	0.98	0.01	0.80	**chr1**	-		
Scaffold_1194	144525903	1.97	0.95	0.03	0.74	**chr1**	+		
Scaffold_100074	146495435	1.95	1.00	0.03	0.70	**chr1**	+		
Scaffold_605	148447732	63.83	0.93	0.22	0.67	**chr1**	-		
Scaffold_2678	212275389	0.37	0.51	0.00	0.62	**chrUN**			
Scaffold_1750	212649219	70.59	0.90	0.23	0.85	**chr1**	+		
Scaffold_100120	283239647	0.25	0.50	1.00	1.00	**chrUN**	+		
Scaffold_653	283486746	0.47	0.52	1.00	1.00	**chrUN**	+		
Scaffold_1948	283960203	8.73	0.97	0.03	0.89	**chr1**	+		
Scaffold_2542	292688886	0.36	1.00	1.00	1.00	**chr1**	+		
Scaffold_2622	1	139.50	0.91	0.52	0.87	**chr2**	-		
Scaffold_100115	139503085	0.33	0.66	0.00	1.00	**chrUN**	+		
Scaffold_100390	139831199	0.10	0.37	0.00	0.96	**chrUN**	+		
Scaffold_588	139935275	0.19	0.95	1.00	1.00	**chr2**	+		
Scaffold_100035	140122483	15.16	0.72	0.05	0.62	**chr2**	-		
Scaffold_100078	155280469	1.39	0.54	0.01	0.49	**chrUN**			
Scaffold_34	156672757	85.80	0.87	0.31	0.78	**chr2**	-		
Scaffold_3074	242468423	17.38	0.83	0.06	0.90	**chr2**	+		
Scaffold_2923	259847680	0.34	0.31	0.00	1.00	**chrUN**			
Scaffold_1820	260183567	0.36	1.00	1.00	1.00	**chr2**	+		
Scaffold_100046	1	9.37	0.91	0.03	0.75	**chr3**	-		
Scaffold_405	9365327	41.21	0.89	0.12	0.81	**chr3**	-		
Scaffold_100109	50572670	0.26	0.26	0.00	0.75	**chrUN**			
Scaffold_1980	50830930	88.32	0.91	0.28	0.41	**chr3**	-	reversed	Reference and optical maps alignments and centromeric repeats at the beginning of the scaffold_1980
Scaffold_3136	139153606	0.47	0.96	0.00	0.86	**chr3**	+		Centromeric repeats at the beginning of the scaffold_3136
Scaffold_2854	139623671	3.56	0.98	0.02	0.49	**chr3**	+		
Scaffold_339	143188428	0.64	0.46	0.00	0.90	**chrUN**	+		
Scaffold_1791	143833524	2.10	0.25	0.00	0.83	**chrUN**	+		
Scaffold_100089	145932556	0.52	1.00	1.00	1.00	**chr3**	-		
Scaffold_391	146447927	167.25	0.92	0.50	0.72	**chr3**	+		

Tables S1 (continued) Grouping, location and orientation confidence scores obtained with RagTag and assignation of the scaffolds to chromosomes 4, 5, 6 and 7

Scaffolds	Positions	Length (Mb)	Grouping confidence	Location confidence	Orientation confidence	Assignation	Orientation	Manual curation	Commentary
Scaffold_1420	1	28.28	0.85	0.12	0.72	**chr4**	-		
Scaffold_503	28276606	111.45	0.83	0.49	0.62	**chr4**	-		
Scaffold_100088	139728581	0.39	0.46	0.00	1.00	**chrUN**	+		
Scaffold_100123	140116163	0.21	0.80	1.00	1.00	**chr4**	+		
Scaffold_2012	140326476	4.57	0.96	0.05	0.90	**chr4**	+		
Scaffold_100021	144893014	29.45	0.80	0.14	0.53	**chr4**	+		
Scaffold_100032	174339204	17.11	0.80	0.09	0.54	**chr4**	-		
Scaffold_100106	191448136	0.28	1.00	1.00	1.00	**chr4**	+		
Scaffold_100051	191725424	7.79	0.52	0.02	0.84	**chrUN**	+		
Scaffold_1644	199517487	2.03	0.48	0.01	1.00	**chrUN**	+		
Scaffold_8135 frag2_42-68Mb	201546798	25.84	0.78	0.11	0.65	**chr4**	-	split	Scaffold_8135 aligned both to the chr4 and chr5
Scaffold_100063	227389902	3.00	0.89	0.02	0.96	**chr4**	+		
Scaffold_8135 frag1_1-42Mb	1	42.33	0.87	0.24	0.54	**chr5**	-	split	Scaffold_8135 aligned both to chr4 and chr5
Scaffold_3415	42330101	2.32	1.00	0.03	0.99	**chr5**	-		
Scaffold_264	44645588	68.28	0.83	0.36	0.84	**chr5**	-		
Scaffold_1218	112928476	57.28	0.91	0.33	0.93	**chr5**	+		
Scaffold_100128	1	0.18	1.00	1.00	1.00	**chr6**	+		
Scaffold_293	177348	9.87	0.95	0.04	0.94	**chr6**	-		
Scaffold_1452	10044232	52.10	0.91	0.20	0.88	**chr6**	-		
Scaffold_100172	62141462	12.94	0.74	0.04	0.67	**chr6**	-	Moved between Scaffold_100025 and 4533	Optical maps alignments
Scaffold_4533	75082935	0.66	0.75	0.01	0.66	**chr6**	-	Moved between Scaffold_100172 and 184	Optical maps alignments
Scaffold_100086	75740204	0.95	0.95	0.04	0.59	**chr6**	-	Moved between Scaffold_100036 and 100025	Optical maps alignments and centromeric repeats all along the Scaffold_100086
Scaffold_852	76689220	76.74	0.86	0.28	0.66	**chr6**	+	Moved between Scaffold_1452 and 100036	
Scaffold_100025	153429261	21.67	0.89	0.09	0.42	**chr6**	-	Moved between Scaffold_100086 and 100172	Centromeric repeats at the beginning of the Scaffold_100025
Scaffold_100036	175096010	15.31	0.95	0.18	0.50	**chr6**	+	Moved between Scaffold_852 and 100086	Centromeric repeats at the end of the Scaffold_100036
Scaffold_184	190410105	67.00	0.84	0.23	0.81	**chr6**	-		
Scaffold_3195	257410051	5.55	0.91	0.03	0.95	**chr6**	-		
Scaffold_1763	262955763	5.22	0.88	0.08	0.90	**chr6**	-		
Scaffold_100102	1	0.29	0.99	0.36	0.63	**chr7**	+		
Scaffold_100055	292087	6.80	0.96	0.07	0.94	**chr7**	+		
Scaffold_1301	7091952	146.59	0.58	0.51	0.71	**chr7**	-		
Scaffold_2567	153684823	105.08	0.61	0.40	0.70	**chr7**	+		
Scaffold_100092	258763135	0.10	1.00	1.00	1.00	**chr7**	-		
Scaffold_3516	258865757	0.38	1.00	1.00	1.00	**chr7**	-		
Scaffold_100127	259248350	0.23	0.62	0.00	1.00	**chrUN**	+		
